# Beyond transparency: architectural application of robotically fabricated polychromatic float glass

**DOI:** 10.1007/s41693-022-00071-6

**Published:** 2022-04-28

**Authors:** Rena Giesecke, Rémy Clemente, Ioanna Mitropoulou, Eleni Skevaki, Christian Thiago Peterhans, Benjamin Dillenburger

**Affiliations:** ETH Zurich, Architecture, Digital Building Technologies, Stefano-Franscini-Platz 1, 8093 Zurich, Switzerland

**Keywords:** Additive manufacturing, Robotic fabrication, Granule deposition, Polychromatic, Glass

## Abstract

This research investigates robotically fabricated polychromatic float glass for architectural applications. Polychromatic glass elements usually require labor-intensive processes or are limited to film applications of secondary materials onto the glass. Previous research employs computer numerical control (CNC) based multi-channel granule deposition to manufacture polychromatic relief glass; however, it is limited in motion, channel control, and design space. To expand the design and fabrication space for the manufacture of mono-material polychromatic glass elements, this paper presents further advancements using a UR robotic arm with an advanced multi-channel dispenser, linear and curved-paths granule deposition, customized color pattern design approaches, and a computational tool for the prediction and rendering of outcomes. A large-scale demonstrator serves as a case study for upscaling. Robotic multi-channel deposition and tailored computational design tools are employed to facilitate a full-scale installation consisting of eighteen large glass panels. Novel optical properties include locally varying color, opacity, and texture filter light and view. The resulting product constructs sublime architectural experiences through light refraction, reflection, color, opacity - beyond mere transparency.

## Introduction

While the float glass market for applications in construction is continuously growing (Statista 2021), traditional custom glass making is a declining industry, and glass artisanship is at risk of extinction (Morris 2021). Despite rapid developments in digital fabrication for a wide range of materials and an excellent potential for the customization of glass elements, digital fabrication methods for glass at the construction scale are still in their infancy. This research implements digital fabrication to fill the gap between mass-produced building elements and custom-crafted glass artifacts.

### Background

In the history of architecture and construction, glass has played a crucial role in creating transparent building skins. In the 13th century BCE, the first polychromatic glass artifacts were manufactured (Wight 2011) and in 100 CE, the Romans installed the first cast small flat glass parts in windows (McGrath and Frost 1937). Only much later, in the 7th century CE, stained glass was developed and found its application in buildings. Stained glass was primarily installed in churches and monasteries and was made by joining differently colored glass pieces with metal joinery (The Metropolitan Museum of Art 2020). Since the 18th century, glass was blown into a cylinder shape and then unfolded into flat sheets for application in buildings. The invention of the Pilkington float glass process in 1952 enabled the production of float glass with standardized quality (Pilkington 1969). Since then, glass is primarily implemented as a standardized, fully transparent material. Architects and engineers have pushed glass technology for maximizing size and transparency, nurturing a building culture of minimal material presence and maximal transparency in the glass (Giesecke and Dillenburger 2022). Meanwhile, multi-colored glass is rarely implemented in construction. This is due to the labor intensiveness of the manual crafting process, which results in high costs and non-standardized quality, and the lack of automated methods for manufacturing polychromatic glass at the construction scale. Ceramic printing and foils enable the customization of mass-produced float glass through surface deposition of secondary material in an automated manner (DipTec 2021). However, ceramic inks and foils remain a surface application that requires fusion with a secondary material, making recycling complicated and producing relief or material textures in glass elements impossible.

### State of the art

Recent developments in additive manufacturing have enabled the customization of building components through robotic fabrication and computational design. For glass, additive manufacturing technologies are still limited to small-scale applications, and digital fabrication methods for uniform glass elements at the construction scale are still in their infancy due to the difficulty of processing both glass and heterogeneous properties in materials. The Massachusetts Institute of Technology (MIT) has developed a process for three-dimensional printing of free-form and multi-colored small-scale glass artifacts, employing an extrusion process in a heated chamber (Klein et al. 2015). The Glass and Transparency Group at TU Delft has produced glass bricks using a kiln-casting method for glass in different colors (Bristogianni et al. 2018). Previous work has demonstrated the potential of utilizing robotic fabrication for grading glass in an automated, controlled process (Michopoulou et al. 2021). Additionally, other research has demonstrated the customization of industry-ready float glass, enabling the inscription of locally varying colors, opacity, and relief into float glass using a CNC setup and a multi-channel tool combined with a pixel-based granule deposition approach (Giesecke and Dillenburger 2022).

### Approach

In this research, we are extending the state of the art by investigating and applying a Universal Robot (UR 10) setup to increase the freedom of tool and motion control beyond existing research; linear and curved path deposition as an advancement of pixel and line deposition methods, and custom computational design and tool path strategies for color pattern generation. Robotic deposition strategies for continuous pattern production along segments and beyond robot reach enables large-scale production. To predict outcomes of the robotic deposition process, a digital tool that visualizes granular deposition results is set up enabling rendering of design iterations prior to the fabrication process. To understand the trade-offs of the presented method and identify further steps of research required for the application of the technology at construction scale, energy consumption and cost of the resulting elements for the product stage are evaluated. A large-scale demonstrator serves as a case study to test the continuity of pattern, large-scale fabrication of a large number of panels, logistics, and installation process.

**Fig. 1:**
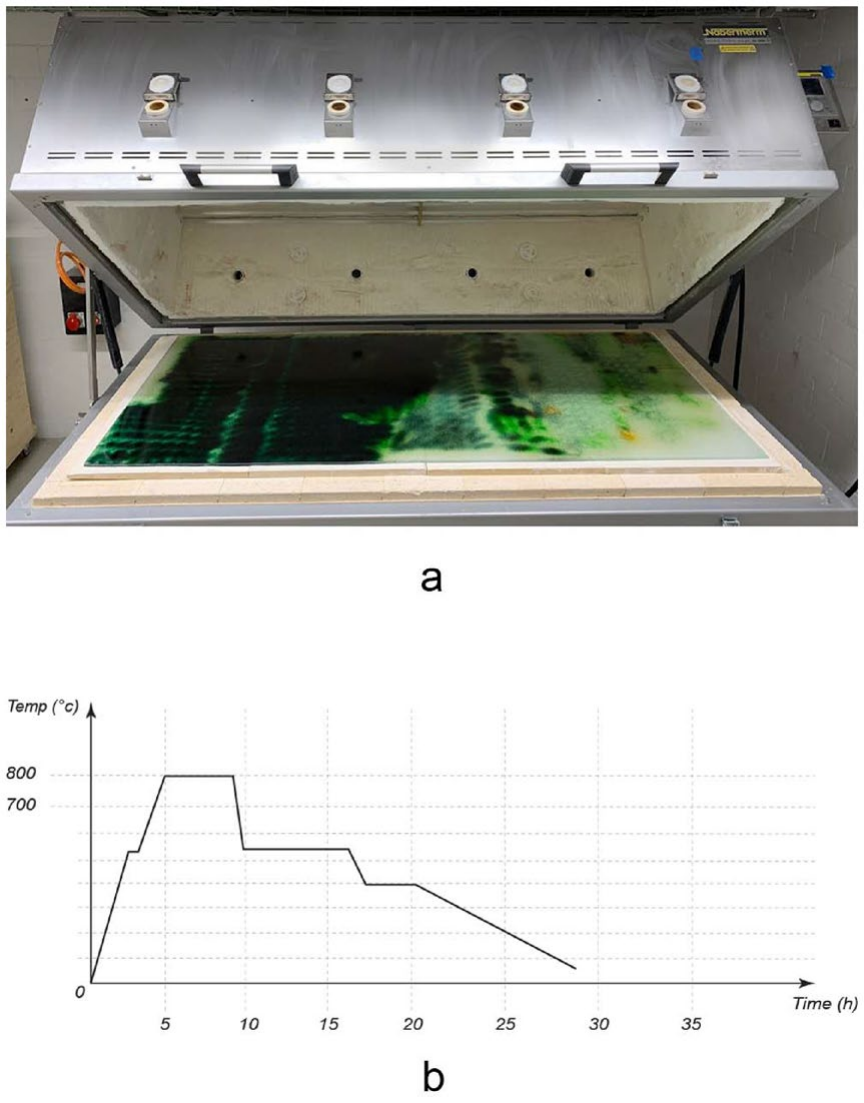
**a** Colored $1 \times 2$ m panel in Nabertherm GF600 kiln. **b** Heat curve for fusing colored granules and float glass

**Fig. 2:**
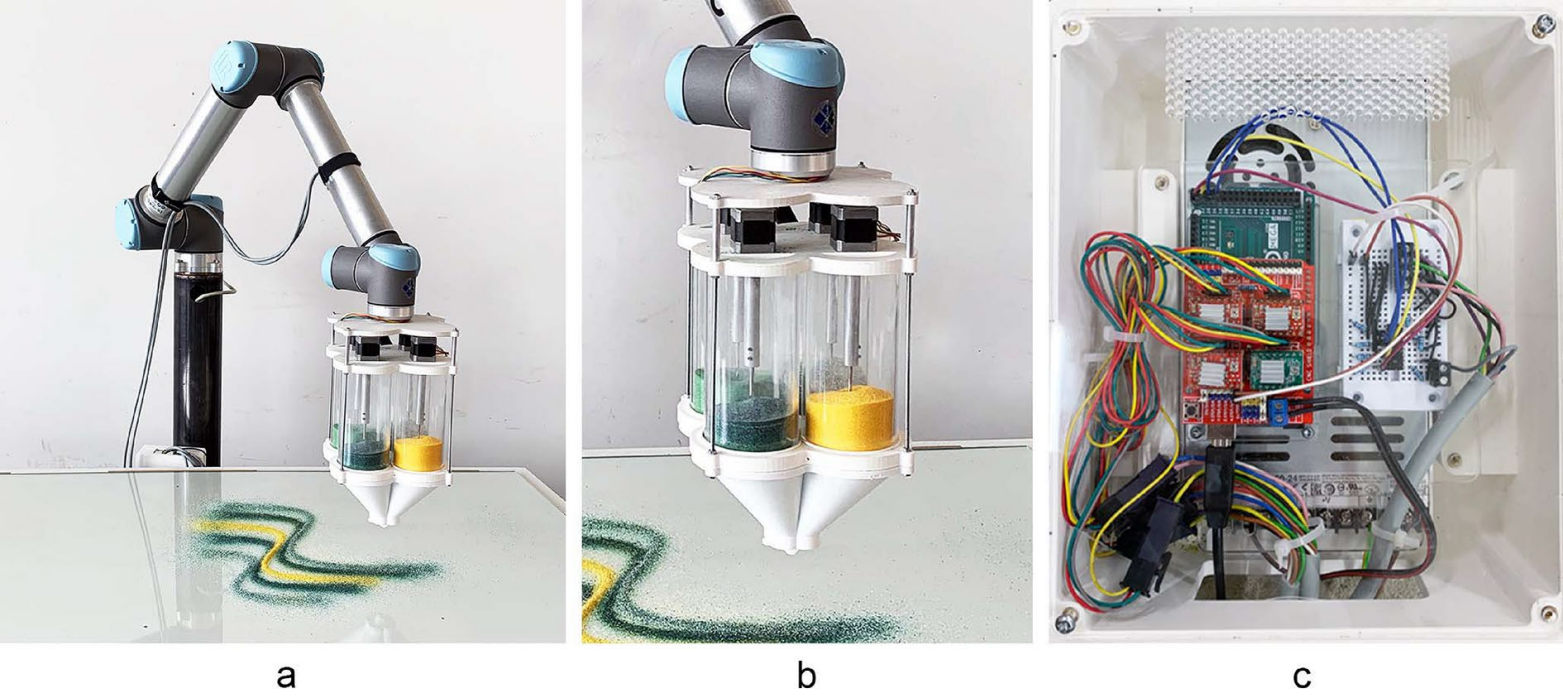
**a** UR10 robotic setup with multi-channel tool. **b** Robotic multi-channel tool head. **c** Arduino Mega equipped with Arduino CNC shield for channel control

## Materials and methods

This section gives an overview of the materials, hardware, and software used in this research. For all experiments, off-the-shelf float glass from St. Gobain with 8mm material thickness is used as base material (St. Gobain 2016). To ensure compatibility between float and granular glass and avoid breakage, granular colored OPTUL Spezialglas K2 is used for all kilning tests, which is specifically developed for fusing with flat glasses (Optul 2021). All kilning experiments are executed in a Nabertherm GF600 glass kiln with $1\times 2 \times 0.4$ m inner volume (Nabertherm 2021). The robotic setup consists of a UR10 robotic arm by Universal Robots. The motors used on the tool are NEMA 17 bipolar stepper motors controlled by a programmed Arduino Mega 2560 microcontroller. Fabrication commands, including robot motion and tool control, were written in URScript and sent to the UR robot using offline communication.

### Kilning process

For the kiln fusing process, the panel is placed in the kiln on top of ceramic plates (Fig. 1a). As ceramic plates are not available in the panel size, a glass fiber textile is placed below to avoid a grid pattern in the glass due to the gaps between ceramic plates. The panel is kilned at peak temperatures of 800 degrees Celsius into a polychromatic uniform glass element. At 800 degrees Celsius, all colors of the OPTUL color palette achieve their final color and melt into smooth coloring without causing a global deformation of the glass panel and its edges. The kilning process can result in slightly rounded edges . The controlled, slow cooling process allows for releasing stresses contained in the glass (Fig. 1b).

**Fig. 3:**
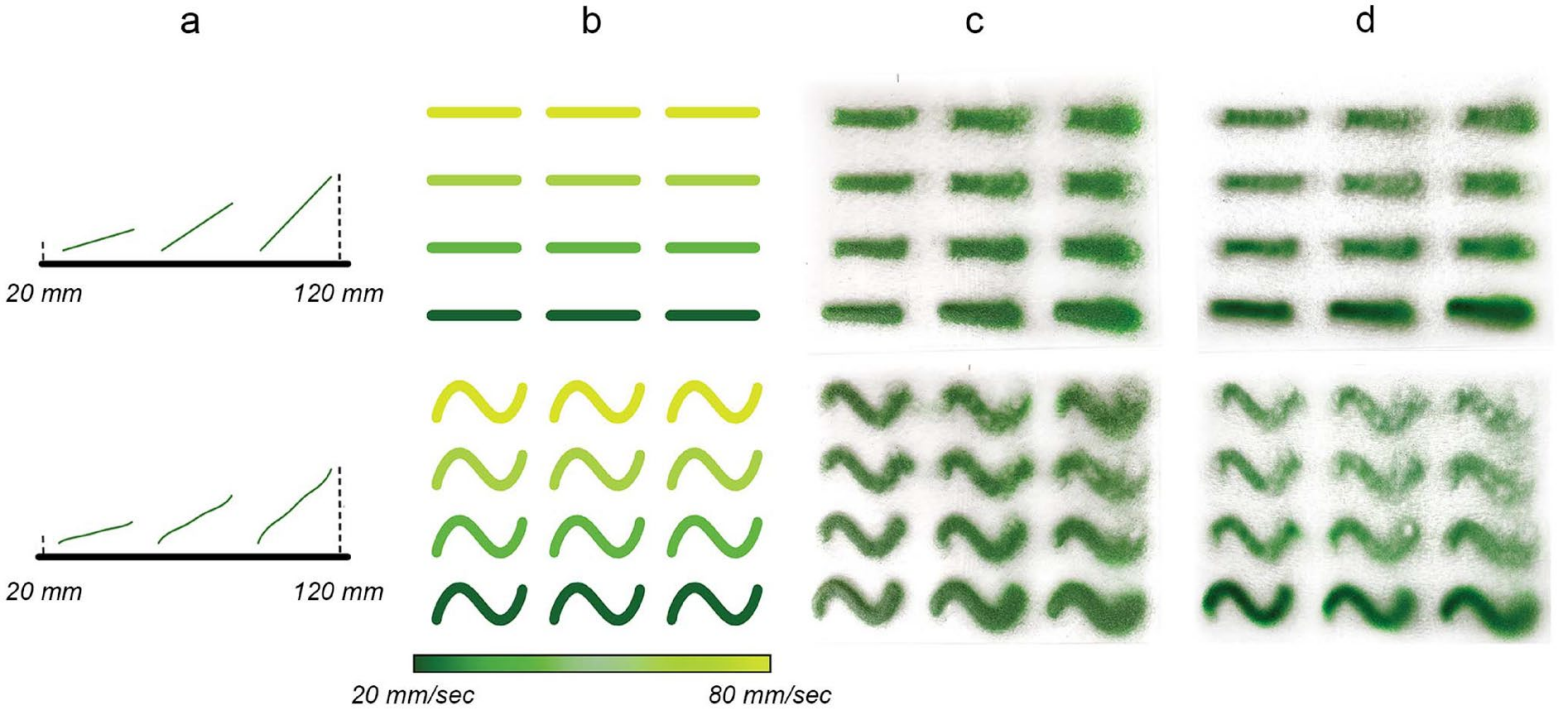
Robotic brush stroke parameters (**a**, **b**) and results (**c**) before (**d**) after kilning depositing granules along a linear (top) and a curved geometry (bottom). Distance range: 20–120 mm. Velocity range (visualized from green to yellow): 20–80 mm/s. Glass panel dimensions: $60 \times 45$ cm

### Robotic multi-channel tool setup

The robotic setup consists of the custom tool head attached to a UR10 robot (Fig. 2a). The multi-channel tool for dispensing granules (Fig. 2b) consists of four independent acrylic color channels, each equipped with one motor. The motors are mounted on a plate, and the motor shafts are connected to metal screws, allowing for rotating positive displacement of the granules from the four channels at a constant predefined speed. The plates holding the motor and connecting to the robotic arm and the bottom funnels are all 3D FDM printed using poly lactic acid (PLA) and are connected with metal screws, reducing the overall weight of the tool while offering enough stability. Motor movement is triggered from the digital outputs (DO) of the UR10. To enable the control of the bipolar stepper motors through the UR’s DOs an electrical interface is established. An Arduino Mega equipped with a Joy-it Arduino CNC shield is mounted with four A 4988 stepper drivers and powered by a 24 Volt power supply (Fig. 2c). Also, a four-channel Vishay optocoupler is used to transfer the Robot’s DO’s signal to the Arduino while protecting its circuit. In terms of software, the Arduino-compatible Accelstepper library (AirSpayce 2010) is used to control the motors’ motion. All of the stepper drivers are set to a full step configuration. Each motor is programmed to react to a step and direction input command triggered by the UR’s DO’s.

**Fig. 4:**
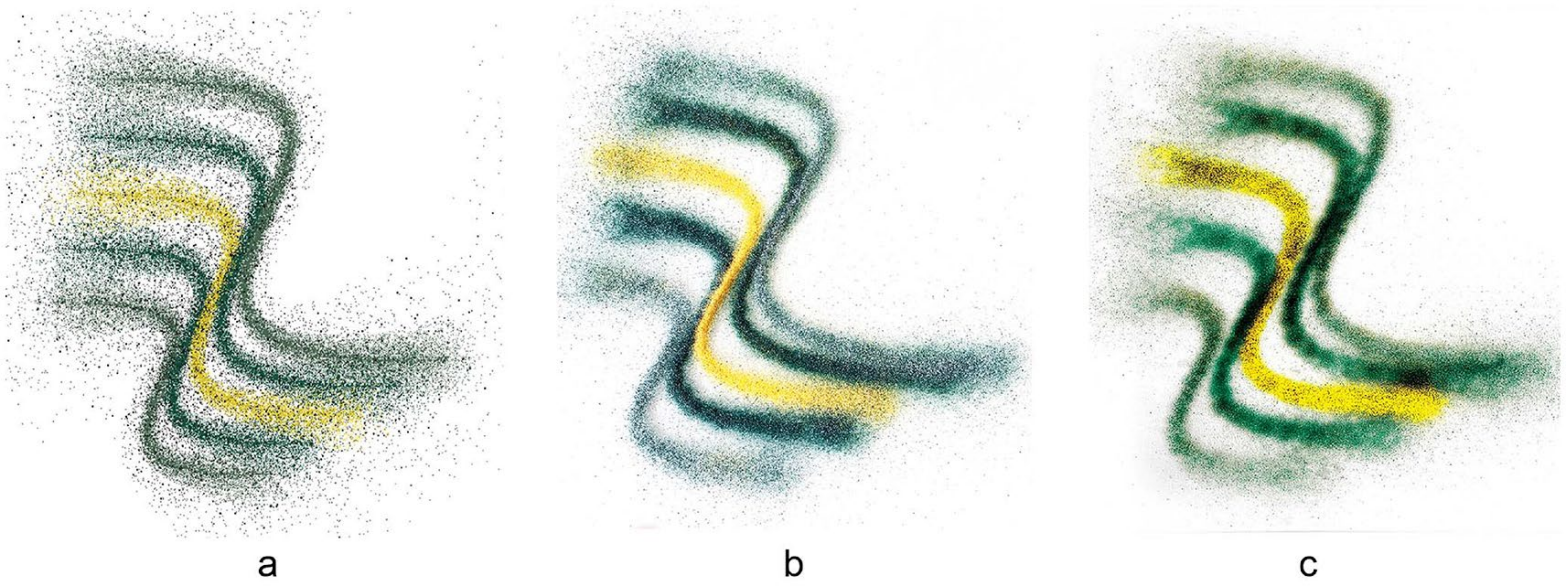
**a** Computationally generated pattern prediction, **b** robotically deposited granules before kilning, **c** after kilning

### Parameter calibration and robotic brush strokes

A series of calibration tests are carried out to determine the effect of the various process parameters of granules disposal on the final coloring effect.

The parameters identified as most meaningful for controlling the process are the following: first, the tool center point (TCP) distance from the glass surface controls the spread of the granules on the glass surface; as the distance increases, the granules scatter more, creating a less dense coloring, while a smaller distance creates an opaque surface.

Second, the robotic linear TCP velocity and tool motors’ angular velocity both have the effect of determining the number of granules that are deposited. To reduce the control parameters, the tool motor’s velocity is kept constant and the robot’s velocity varied instead.

Figure 3 displays the calibration tests performed to quantify the effect of those parameters and determine the thresholds within which the repeatability of the results is acceptable for our application. We use a velocity range of 20–80 mm/s and a vertical distance range of 20–120 mm in the tests. A notable observation is that when depositing from a higher distance, often at the beginning and the end of paths, undesirable discontinuities are observed in the distribution of granules due to them bouncing when they meet the glass. In addition, higher distances from the surface can lead to the blurriness of robotic brush strokes or even merging when their center lines are close. These tests served as a basis for controlling the parameters of granules deposition in the later steps.

### Pattern prediction

A computational workflow can significantly aid the creation of a design process for a digitally fabricated artifact for approximately visualizing the result so that the need for physical prototypes is reduced. Such a workflow, which we call the pattern prediction, facilitates and accelerates the design process and can save material and energy by reducing the number of physical prototypes. Since the aim of the pattern prediction is to be helpful as a design tool, we opt for a quick and lightweight workflow to the expense of it being physically accurate. This trade-off between speed and accuracy is justifiable in a design process, where the need for quick design iterations surpasses the need for high accuracy of the visual feedback.

**Fig. 5:**
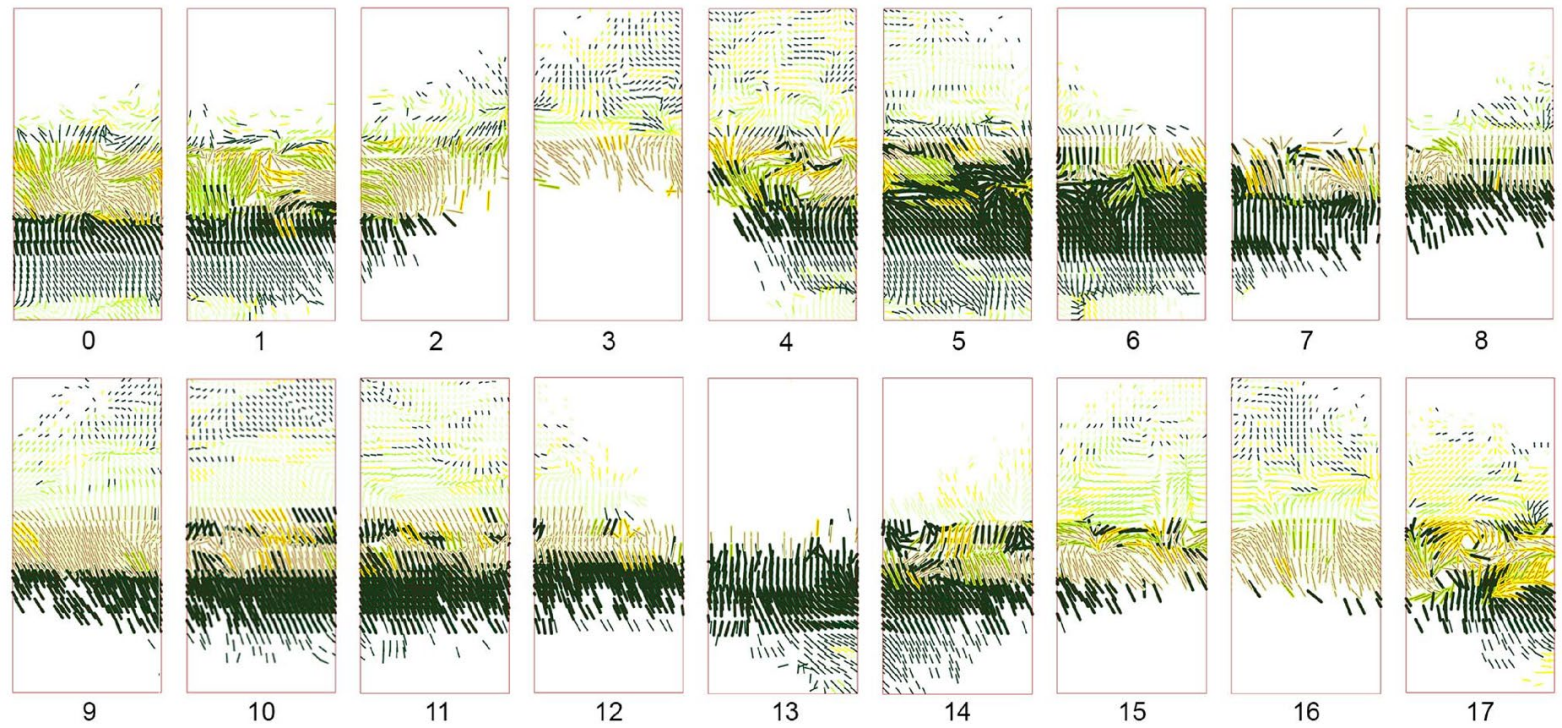
Color pattern design of 18 panels represented by lines

The two parts of the fabrication process that determine the final result are; the deposition of granules and their scattering on the glass surface and the kilning process, which fuses granules and float glass. Instead of simulating the behavior of hundreds of thousands of granules landing on the surface and then the process of glass kilning, we opt for a simple pixel-based approach. Starting with a white canvas whose resolution matches the smallest granules’ size, pixels (equivalent to granules) around the centerline of the robot’s deposition path are colored using a random distribution. The physical calibration tests (Fig. 3) are used to fine-tune the visual scattering and density of the digitally pixelated granules to generate images close to the results of the physical tests. The pattern prediction takes as input the fabrication commands destined for the robot and instantly generates a faithful image of the expected pattern (Fig. 4a), which can then be used for rendering on any commercial software (Fig. 5), facilitating design iterations. Figure 4b shows the actual granule distribution after printing and Fig. 4c the fused granules after kilning. The pattern prediction strategy is implemented in Python, using the matplotlib library (Hunter 2007). The implementation of this functionality can be accessed online (Mitropoulou 2021).

### Production times, cost and energy consumption

This section gives an overview of production times, energy consumption and production costs to better understand the impact and trade-offs of the presented robotic fabrication method compared to off-the-shelf float glass.

#### Production times

The granule deposition time per square meter of the fully colored glass surface is approximately 40 min. Depending on the toolpath strategy applied and the coloring intensity, the print time can slightly vary with a range of 50–80 min printing time per panel. Traveling and extrusion speed are highly dependent on the robotic process parameters and quantity of granules used.

#### Equipment costs

**Table 1:** Energy consumption and pane﻿l cost

Glass technology	Color volume	Energy consumption	Additional cost	Total price
Float glass clear (8 mm)	None	101 $\frac{\mathrm{kWh}}{\mathrm{m}^2}$ (St. Gobain 2016)	None	50 $\frac{\mathrm{Euro}}{\mathrm{m}^2}$ (sales price)
Robotically fabricated polychromatic float glass (8mm)	2.2 kg OPTUL Spezialglas	Float glass production: 101 $\frac{\mathrm{kWh}}{\mathrm{m}^2}$ (St. Gobain 2016) Granule deposition: 67.5 $\frac{\mathrm{kWh}}{\mathrm{m}^2}$ (Clear Path Robotics 2021) Kilning: 50 $\frac{\mathrm{kWh}}{\mathrm{m}^2}$	Float glass: 50 $\frac{\mathrm{Euro}}{\mathrm{m}^2}$ 2.2 kg $\times$ 20$\frac{\mathrm{Euro}}{\mathrm{kg}}$ = 44 $\frac{\mathrm{Euro}}{\mathrm{m}^2}$ (Optul 2021) Granule deposition: 10.12 $\frac{\mathrm{Euro}}{\mathrm{m}^2}$ Kilning: 7.50 $\frac{\mathrm{Euro}}{\mathrm{m}^2}$	111.62 $\frac{\mathrm{Euro}}{\mathrm{m}^2}$ (production price excl. machine cost)

The costs for the multi-channel tool head are composed of the costs for off-the-shelf acrylic pipes, standard metal drill bits, and 3D printed poly lactic acid (PLA) parts. The toolhead provides a low-cost Do-It-Yourself solution at approximately 100 Euro hardware cost for the tool body and around 200 Euro for the electronics and motors, resulting in an overall one-time investment of 300 Euro. Robotic arms or CNC setup are not specific to the presented fabrication method, vary significantly by brand, thus are not separately specified in this cost overview. The granular glass raw material adds 44 Euro/m$^2$ cost for coloring one entire panel. Glass kiln prices can vary by country, brand, and quality.

#### Energy consumption and panel cost

This section investigates the energy consumption and cost of the process compared to conventional float glass to understand the trade-offs of this method for practice. Table 1 provides an overview of the costs and energy consumption per m$^{2}$ of material produced. While the average energy consumption for float glass of 8mm thickness is 101 kWh/m$^{2}$, robotically fabricated glass requires an additional 117.5 kWh/m$^{2}$. This additional energy consumption is composed of 50 kWh/m$^{2}$ for fusing and 67.5 kWh/m$^{2}$ for robotic dispensing (Table 1). Thus, the required energy for robotically fabricated polychromatic glass increases by 115% compared to conventional float glass. The cost increase is based on the cost for this additional energy and the glass granules which can be approximated at 2.2 kg/m$^{2}$. The total cost per m$^{2}$ excluding machine cost increases by 122% compared to conventional float glass. For comparison, information on the energy consumption and costs for alternative coloring processes for glass such as digital ceramic printing and silkscreen printing was also requested, however no sufficient data could be provided.

## Large-scale demonstrator

A large-scale demonstrator serves as a case study for upscaling the presented technology. The pavilion is a forty square meter installation with a footprint of $4 \times 6$ m and 2.5 height consisting of eighteen individually designed glass elements of $2 \times 1$ m size assembled on an oval floor plan. The polychromatic glass screen has an overall surface of 36 m$^2$ and is held by a CNC milled wood-steel frame. All eighteen glass panels for the pavilion were robotically printed and kilned in-house within 36 days. The overall design-to-production time was eleven weeks, with an on-site assembly time of 2 days. The following sections provide an overview from the design to construction of the large-scale demonstrator.

### Site data as design input

Among other color patterning approaches explored, the color and transparency patterns for the large-scale demonstrator are designed in relation to the site. This coloring approach aims to construct a heterogeneous material filter for light and view, targeting the superposition of the colored glass and the existing site. For this purpose site data is captured with the aid of a FARO Focus X330 scanner to provide context for the computational generation of pattern iterations. After the scanning operation, the scan data is converted to a point cloud through Autodesk Recap (Autodesk 2020) and filtered inside Cloud Compare (Open Source Project 2021). The goal is to establish a direct relationship between the granular nature of the material and the point cloud representation that the 3D scan provides. After encoding the site to a set of 200 million coordinates and RGB values, projection operations imprint the site data onto the glass panels of the pavilion as 2D images.

### Computational color pattern design

To create a rich and highly differentiated color pattern, a series of digital filters are deployed that translate and transform the captured site data.

**Fig. 6:**
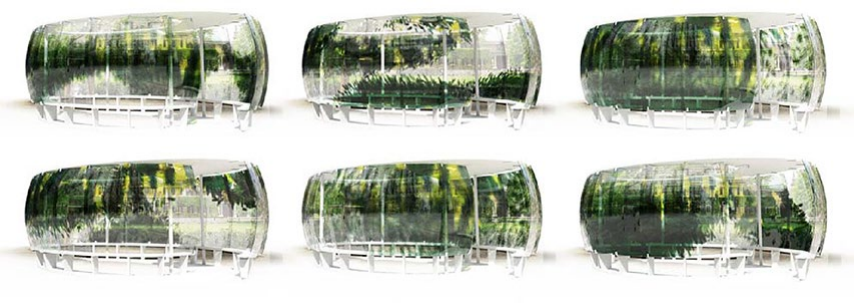
Rendering of exemplary color iterations for the full-scale installation

First, the boundaries between different elements of the scene are identified using an edge detection algorithm (Sobel filter) implemented in the Python Scikit library (Pedregosa et al. 2011). The resulting edge map is used to generate the Gradient Vector Flow (GVF) of the image (Xu and Prince 1998): a set of vectors anchored to a two-dimensional grid of points (*x*, *y*) on the domain of a panel, pointing towards the boundaries. The four directions parallel to the coordinate axes correspond to the four available colors of granules. Colors are assigned to the vector field by calculating which of those four directions has the smallest angle with each vector. Subsequently, the vectors are unitized, and magnitudes are assigned based on the y coordinate of the vectors’ anchor point. This results in a gradation that peaks at eye level and fades towards the bottom and top of the panels (Fig. 5). On the global pattern scale, certain regions are masked to allow for site fragments to be directly visible through the glass. A series of variations exploring linear or diagonal development of color and transparency are generated and evaluated. The aim is to maximize the diversity of the experience, designed as a succession of intense color areas, graded opacity, and complete absence of color (Fig. 6).

### Robotic tool path and fabrication workflow

**Fig. 7:**
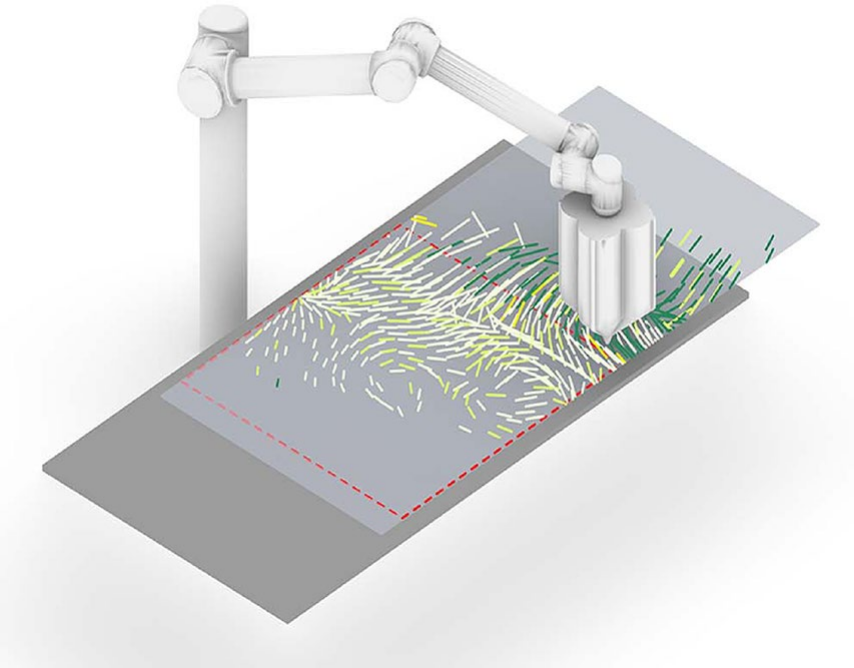
Print surface segmentation for full panel coverage within UR10 robot reach

**Fig. 8:**
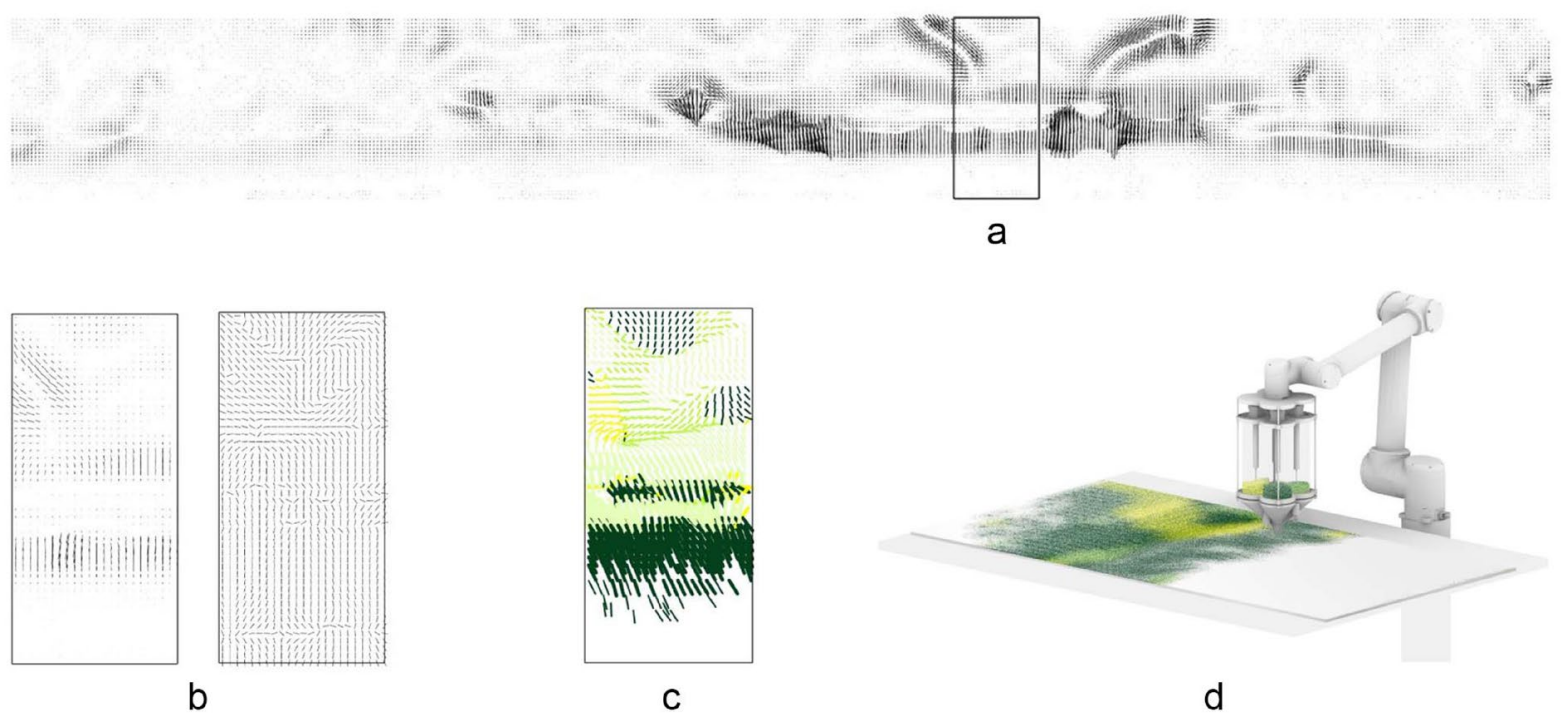
**a** Creation of panels for fabrication from the vector map, **b** the gradient vector flow, **c** brush definition, **d** fabrication setup

For the translation of the pattern into a robotic tool path for the fabrication of panels, each panel-specific file is converted into a JSON file containing the brush strokes with the following data:

- start and endpoints of the line segments extracted from the vectors’ directions and magnitudes,
- robot speeds for the execution of each line,
- and colors selected from the end effector’s four channels.

To avoid mounds of granules and create a readable pattern, a threshold is defined for the minimum length of a line below, which is reduced to a single dot. Variations in velocity and distance are applied to the remaining lines, resulting in a progressive variation of blurriness and line thickness. Every production file is then divided into two parts since the robot’s reach cannot cover an entire panel (Fig. 7). Before sending the information to the robot, a sorting algorithm rearranges the order of line segments to minimize traveling time.

After the granules are deposited on the first half of the panel, the panel is manually slid for the production of the second half. Deposited granules are steady enough to stay in place during the process. Figure 8 summarizes the three major phases of the digital to physical process, from data input to fabrication output.

### Logistics, assembly, and maintenance

**Fig. 9:**
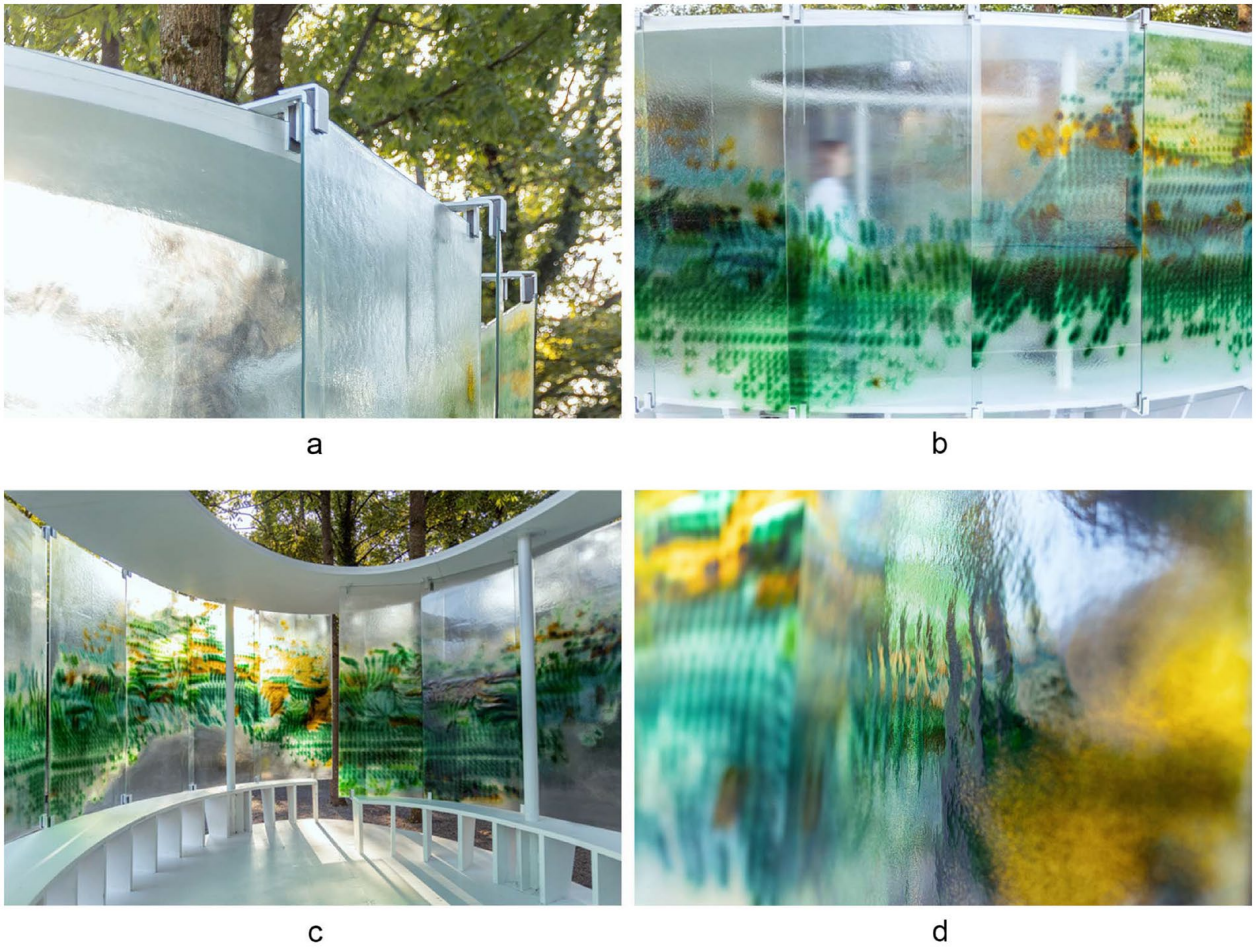
**a** Metallic clamps holding the glass panels, **b** elevation of polychromatic panels installed installation, **c** inside-out view through glass, **d** detail of glass relief resulting in reflections

Logistics, assembly, and maintenance differ slightly from procedures for off-the-shelf float glass applications. If the material relief imprinted into the glass panels is almost flat, the panels can be stored and transported on standard glass transport racks. In case of a more intense texture or relief, panels can be wrapped into bubble wrap and transported in the same manner to the construction site. The glass is lifted with vacuum suckers for the installation process and installed using punctual metal clamps with a soft foam inlay. Metal clamps with soft foam inlays hold each panel on four points (Fig. 9a). The panels are designed and installed with an overlap of 5–7 cm to provide a continuous pattern appearance despite gaps in between (Fig. 9b). Shades of green and yellow echo the site’s color palette and establish moments where the surrounding garden and glass color pattern superimpose in manifold ways. Varying light conditions activate the polychromatic glass screen in different ways over the day and seasons (Fig. 9c). The glass relief creates effects of reflection (Fig. 9d).

This approach enables a stable and straightforward installation on-site while also allowing for tolerances caused by the slight deformation of the glass corners during the kilning process. For cleaning the glass panels, standard manual cleaning procedures can be applied. However, automated cleaning methods would not be applicable in locations where relief is present.

Challenges that were observed during the demonstrator fabrication and assembly include the challenge of installing panels with deformed edges, the milkiness of some panels resulting from dust contamination and the long kilning times of panels.

## Conclusions

This paper demonstrates that further advancing robotic setup, custom computational design, and prediction tools, collecting information on energy consumption and costs enables the construction of a large-scale demonstrator with a solid design-to-production workflow. The following paragraphs provide a detailed description of the contributions made:

*Robotic setup and multi-channel tool:* The Universal Robotic UR10 arm and multi-channel setup present a novel setup compared to the CNC-based approach presented in previous research. The six axes of the robotic setup unfold a wide range of motion freedom and channel control. With the novel robotic approach, a universal approach for the fabrication of polychromatic glass is provided, motors can potentially be run simultaneously, and the additional degree of freedom can be used for creative explorations.

*Toolpath and parameter calibration:* Novel toolpath approaches include linear and curved robotic brush strokes. These open up a range of design options beyond a pixel-based approach. Robotic process parameters become a creative tool for variation. The tests performed also prove fabrication process repeatability with only minimal deviations.

*Pattern Prediction:* The custom pattern prediction presented in this research enables the accurate-enough production of visual outputs of the coloring process. This step is crucial for creating quick design iterations for the proposed fabrication system. Experiments above demonstrate that the minimal inaccuracies of the pattern prediction do not pose an obstacle to its role as a design tool.

*Custom computational tools for pattern design:* Implementing site data as design input puts forward a new approach to the computational design of glass elements, its global color patterns, and transparencies. The presented fabrication workflow enables the translation from pattern design to robotic toolpath, including process parameters that implement different robotic brush strokes. The exemplary site-specific pattern design approach is transferable to other sites and scenarios. Filtering mechanisms through color could be customized concerning architectural requirements for the design of polychromatic panels.

*Cost and energy consumption:* Cost and energy consumption calculations show that the color customization with the presented process at the prototyping stages increases the energy consumption by 115% and cost by 122% compared to fully-transparent standard float glass. It is relevant to understand the economic constraints and energy consumption of this customization method to evaluate the trade-offs of this method for building practice and compare competing products and technologies.

## Discussion

*Digital crafting of glass for construction:* Digital crafting of glass enables the inscription of novel properties and functions into glass beyond mere transparency and beyond those of a standardized mass product. For the first time, this research enables the fabrication of robotically fabricated polychromatic glass resulting in a large-scale demonstrator.

*Repeatability and predictability:* Implementing robotic tooling for dispensing glass granules opens up the questions of manual versus digital craft, material language, repeatability, and predictability. Robotic dispensing enables the production of repeatable results with only minor deviations while producing a material language and aesthetics controlled through robotic process parameters. Due to the granular nature of the input material, results are similar but never perfectly the same. Material language and process parameters become part of the robotic design space and form a digital crafting language.

*Novel design space and complexity:* The presented method opens up a new design and fabrication space for glass to architects and engineers. The wide range of parameters for design—colors, opacities, textures, computational pattern design, and process parameters - unfolds a new form of complexity in informed and heterogeneous materials that can only be managed and fully explored with custom computational design and fabrication tools. This potential for heterogeneous materials in construction will require novel ways of modeling and thinking of the granular properties of architecture per se.

*Upscaling:* Upscaling from prototype to construction scale brings new challenges in logistics, structural requirements, material parameters, joinery of parts, and pattern continuity. Pattern continuity is enabled by a controlled shifting of the glass panel and pattern segmentation, taking into account robotic reach. However, despite the larger fabrication and kilning setup needed, no fundamental up-scaling complications arise for the panel production process.

*Limitations and challenges:* The fabrication process is linked to specific characteristics in resolution, sharpness and precision, and types of textures that can be produced. Granularity, robotic motion, and melting process in the kiln result in a particular material language. The parameter calibration tests present a range of those, but resolution, scale, and geometry limitations must be investigated in further experiments. Challenges in the production process include the repeatability of kilning results, specifically concerning the transparency of kilned glass panels. Dust contamination in some panels produces milky results caused by dust in the lab environment or the grinding of glass granules in the tool. It remains an open question how kilned colored float glass behaves structurally compared to off-the-shelf float glass; furthermore, the effect of deformations on the edges of the glass pane will have to be investigated.

*Outlook and future work:* Future work will investigate the performance of results with quantitative methods, such as lab testing of structural and optical properties and long-term performance, to enable the application of robotically fabricated polychromatic glass for building projects. Further investigations could include the construction of hermetically closed facades with the produced panels. New approaches to pattern design and tool path generation for polychromatic glass could enable more sophisticated expressions in glass. And further investigations of three-dimensional relief could produce novel material expressions in colored glass architectures.
